# *Tanaella quintanai*, a new deep-water tanaellid (Crustacea: Peracarida: Tanaidacea) from the Colombian Caribbean Coast, with a key to the species of the genus *Tanaella* Norman & Stebbing, 1886

**DOI:** 10.7717/peerj.7571

**Published:** 2019-09-24

**Authors:** Andrés G. Morales-Núñez, Néstor E. Ardila

**Affiliations:** 1 NSF—CREST Center for the Integrated Study of Coastal Ecosystem Processes and Dynamics in the Mid-Atlantic Region (CISCEP), Department of Natural Sciences, University of Maryland Eastern Shore, Princess Anne, Maryland, United States of America; 2 División de Biología Marina, ECOMAR Consultoría Ambiental, Bogotá, Colombia

**Keywords:** Tanaidacea, Tanaellidae, Caribbean region, Deep-sea, *Tanaella quintanai*, Colombia

## Abstract

A new tanaidacean, *Tanaella quintanai* sp. nov., is described based on specimens collected from depths of 1,598 to 2,853 m during 2014–2015. The new species appears to be most closely related to the western Atlantic species, *T. kroyeri* and *T. mclellandi*. *Tanaella quintanai* can be separated from the two former, as well as from the other members of the genus by a combination of characters, including (1) a *labium* with apical lobe bearing one blunt seta (2) a cheliped with the inner margin of the dactylus bearing a sub-proximal bipinnate seta, (3) pereopods 1−3 with basis having sub-dorsoproximal and sub-ventroproximal margins setulose, (4) pereopods 4−6 with basis having ventroproximal margin setulose, (5) pereopods 4−6 with unguis bearing two parallel rows of small setules, and (6) a pleotelson as long as pleonites 1–5 combined. A key separating the currently recognized species of *Tanaella* is presented.

## Introduction

The Tanaidacean fauna from Colombia has received minimal attention thus far. Recently, Morales-Núñez & Ardila (2018) reported the first record for the family Tanaellidae Larsen & Wilson, 2002 in the Colombian Caribbean region. In addition, Morales-Núñez, Morales-Ruiz & Ardila (2017) described a new sphyrapodid tanaidacean, *Sphyrapus caribensis* Morales-Núñez, Morales-Ruiz & Ardila, 2017, reported *Kudinopasternakia siegi* (Viskup & Heard, 1989), and presented detailed information about the species of tanaidaceans that had been previously reported in the Caribbean and Pacific Colombian coasts.

This publication is the second in a series dealing with the Tanaidacea from the Colombian Caribbean region, and it presents a description of a new species of *Tanaella* Norman & Stebbing, 1886.

The family Tanaellidae was established by Larsen & Wilson (2002) and currently contains five genera (i.e., *Araphura* Bird & Holdich, 1984; *Arhaphuroides* Sieg, 1986; *Arthrura* Kudinova-Pasternak, 1966; *Inconnivus* Błażewicz-Paszkowycz & Bamber, 2012; and *Tanaella* Norman & Stebbing, 1886). An examination of tanaidaceans collected during explorations along the outer shelf, continental slope, and continental margin of the Caribbean coast of Colombia during cruises in 2014–2015, revealed the presence of an apparently undescribed species belonging to the tanaellid genus *Tanaella*. The genus was originally erected by Norman & Stebbing (1886) to receive the new species *T. unguicillata* Norman & Stebbing, 1886. *Tanaella* has a cosmopolitan distribution and is recorded over a wide bathymetric range, from shallow waters (38 m) down to abyssal depths (5,450 m) (Guerrero-Kommritz & Błażewicz-Paszkowycz, 2004; Larsen & Heard, 2004; Drumm & Bird, 2016).

## Materials & Methods

**Figure 1:**
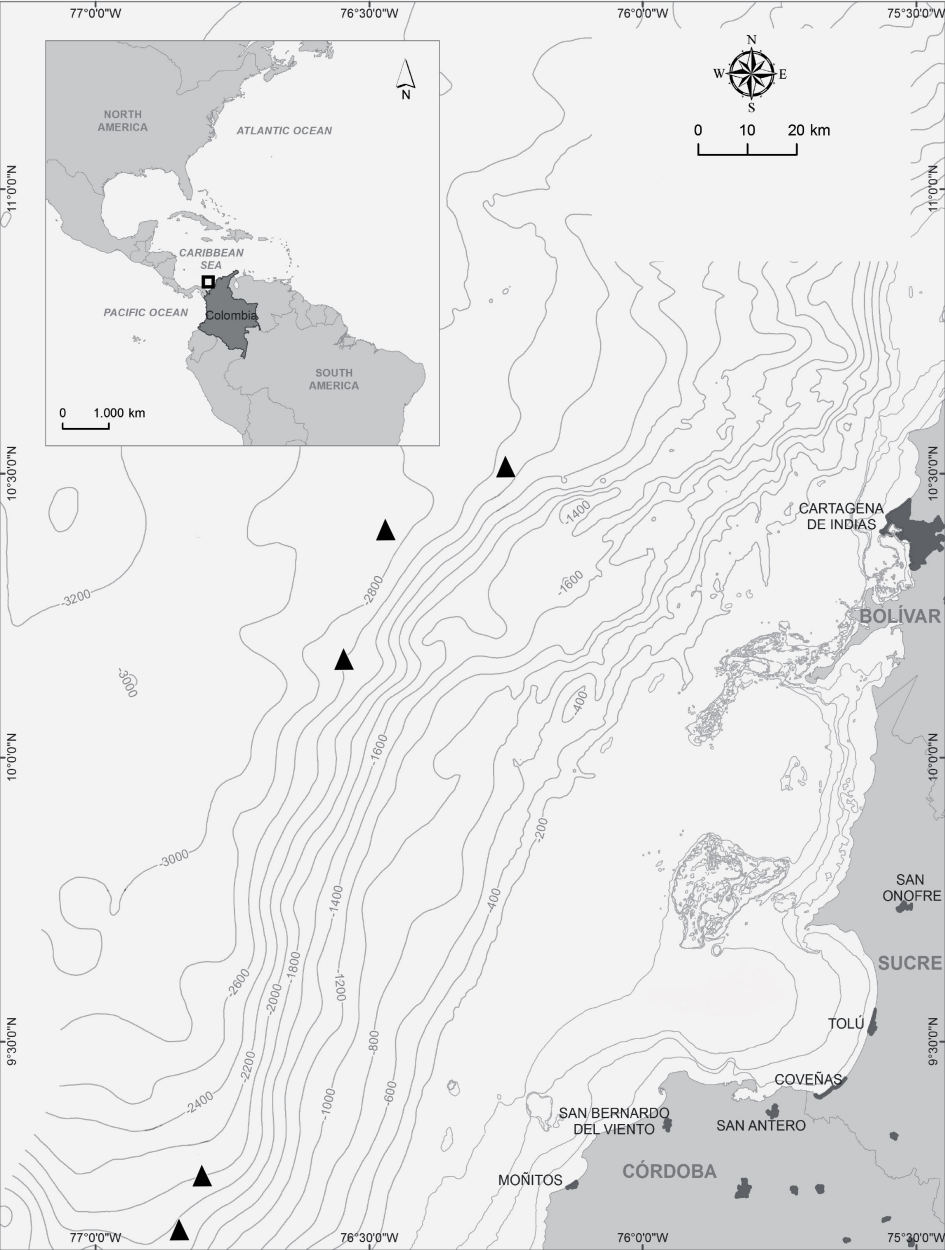
Local distribution. Map of study area, indicating the sampling stations where *Tanaella quintanai* sp. nov., was found.

Specimens were collected using a box corer of 0.25 m^2^ during cruises, aboard the R/V *Proteus* and R/V *Don Rodrigo-B*, working off the southwestern Caribbean Sea of Colombia at depths of 1,598–2,821 m (Fig. 1). Tanaidaceans were sorted, fixed in 6% formalin, and subsequently stored in 70% ethanol. Collection permits were granted by the National Authority of Environmental Licenses—ANLA (FSD, ANLA No. 0723 de 2012; FND, ANLA 1016 de 2012; PAC, ANLA No. 0880 de 2014).

Specimens were dissected under an Olympus SXZ-16 stereomicroscope. Appendages were mounted on glass slides in glycerine and observed with an Olympus BX41 microscope, and drawings were made with a *camera lucida*. Illustrations were prepared with Adobe Illustrator CC (2019) and figures with Photoshop CC (2019). Photographs were taken using an Olympus DP73 digital camera mounted on a stereomicroscope and all specimens were measured with CellSens Dimension 1.11 Imaging Software (Olympus). Maps were created using ArcGIS 10.4.1 software (University of Maryland Eastern Shore (UMES)).

All specimens of *Tanaella* sp. were measured and classified into two life-stages categories (Table 1).

Type material has been deposited in the “Centro de Colecciones Biológicas, Universidad del Magdalena (CBUMAG)”, Santa Marta, Colombia. All measurements were taken in millimeters (mm). Total body length was measured from the tip of the rostrum to the tip of the pleotelson. Terminology generally follows that of Larsen (2003); however, the term “PSS” is here applied for delicate plumose sensory setae found on antennules, antennae, pereopods and uropods (Bird, 2011).

The electronic version of this article in Portable Document Format (PDF) will represent a published work according to the International Commission on Zoological Nomenclature (ICZN), and hence the new names contained in the electronic version are effectively published under that Code from the electronic edition alone. This published work and the nomenclatural acts it contains have been registered in ZooBank, the online registration system for the ICZN. The ZooBank LSIDs (Life Science Identifiers) can be resolved and the associated information viewed through any standard web browser by appending the LSID to the prefix http://zoobank.org/. The LSID for this publication is: [urn:lsid:zoobank.org:pub:BB577909-6C9F-4008-AA2D-2330525FAACE]. The online version of this work is archived and available from the following digital repositories: PeerJ, PubMed Central and CLOCKSS.

**Table 1:** Morphological characters within *Tanaella quintanai*. TL and length of some morphological characters within *Tanaella quintanai* sp. nov. population.

**Stages**	**TL (mm)**	**Pleonites 1–5 combined length (mm)**	**Pleotelson length (mm)**	**Uropod length (mm)**	**Pleonites 1–5 combined/pleotelson ratio**	**Uropod/ pleotelson ratio**
**Non-ovigerous females**						
1 (CBUMAG:MAC:01680)	2.2	0.29	0.26	0.19	1.1	0.70
2 (Dissected, CBUMAG:MAC:01681)	2.3	0.28	0.30	0.23	0.9	0.80
3 (Damaged, CBUMAG:MAC:01683)	2.4	0.26	0.25	0.19	1.0	0.80
4 (Holotype, CBUMAG:MAC:01679)	2.6	0.31	0.29	0.18	1.1	0.60
**Female with marsupium**						
1 (CBUMAG:MAC:01682)	2.2	0.28	0.26	0.20	1.1	0.80

## Systematics

**Table:** 

**Order Tanaidacea Dana, 1849**
**Suborder Tanaidomorpha Sieg, 1980**
**Superfamily Paratanaoidea Lang, 1949**
**Family Tanaellidae Larsen & Wilson, 2002**
**Genus *Tanaella*Norman & Stebbing, 1886**

**Diagnosis.** See Larsen (2005).

**Type-species.** *Tanaella unguicillata* Norman & Stebbing, 1886

**Species.** *Tanaella busteri* Drumm & Bird, 2016*; T. dongo* Bamber, 2005; *T. eltaninae* Guerrero-Kommritz & Błażewicz-Paszkowycz, 2004; *T. forcifera* (Lang, 1968); *T. kimi* Guerrero-Kommritz & Błażewicz-Paszkowycz, 2004; *T. kommritzia* Larsen & Shimomura, 2007; *T. kroyeri* Larsen, Araújo-Silva & Coelho, 2009; *T. mclellandi* Larsen & Heard, 2004; *T. ochracea* Hansen, 1913; *T. paraforcifera* (Lang, 1968); *T. profunda* Guerrero-Kommritz & Błażewicz-Paszkowycz, 2004; *T. prolixcauda* Larsen & Heard, 2004; *T. propinquus* Dojiri & Sieg, 1997; *T. quintanai* **sp. nov**.; *T. rotundicephala* Sieg, 1986; *T. tuberculata* Kudinova-Pasternak, 1989; *T. unguicillata* Norman & Stebbing, 1886; *T. unisetosa* Sieg, 1986.

**Table:** 

***Tanaella eltaninae*Guerrero-Kommritz & Błażewicz-Paszkowycz, 2004**

**Amended diagnosis.** *Female*. Uropod shorter than pleotelson

**Remarks.** In the original description of *Tanaella eltaninae*, Guerrero-Kommritz & Błażewicz-Paszkowycz (2004) stated that this species has an “*Uropod as long as pleotelson*” (p. 3; diagnosis section). However, as it can be observed in the original illustration (p. 4; figs. 1A–1B), the female has an uropod shorter than pleotelson i.e., uropod about 2/3 as long as pleotelson, instead of the uropod being as long as the pleotelson.

**Table:** 

***Tanaella kimi*Guerrero-Kommritz & Błażewicz-Paszkowycz, 2004**

**Amended diagnosis.** *Male and Female*. Uropod shorter than pleotelson

**Remarks.** In the original description of *Tanaella kimi*, Guerrero-Kommritz & Błażewicz-Paszkowycz (2004) stated that this species has an “*Uropod as long as pleotelson*” (p. 8; diagnosis section), which was based on a male (holotype). However, as it can be observed in the original illustrations of a male (p. 9; figs. 4A–4B) and female (p. 9; fig. 4F), the male and female have an uropod shorter than pleotelson i.e., uropod about 2/3 as long as pleotelson, instead of the uropod being as long as pleotelson.

**Table:** 

***Tanaella quintanai* Morales-Núñez sp. nov.**
urn:lsid:zoobank.org:act:C9C6AD95-01BB-46F8-92D8-426916C451AD
(Figs. 2–9)

**Synonyms:** *Tanaella* sp. Morales-Núñez & Ardila, 2018

**Material examined.** *Holotype*—non-ovigerous ♀ (CBUMAG:MAC:01679), TL 2.6 mm, Station (Stn) E05PAC (9°14′3.8″N–76°47′14.57″W), depth 1,598 m, substrata: “muddy bottom”, 27-July-2014.

**Figure 2:**
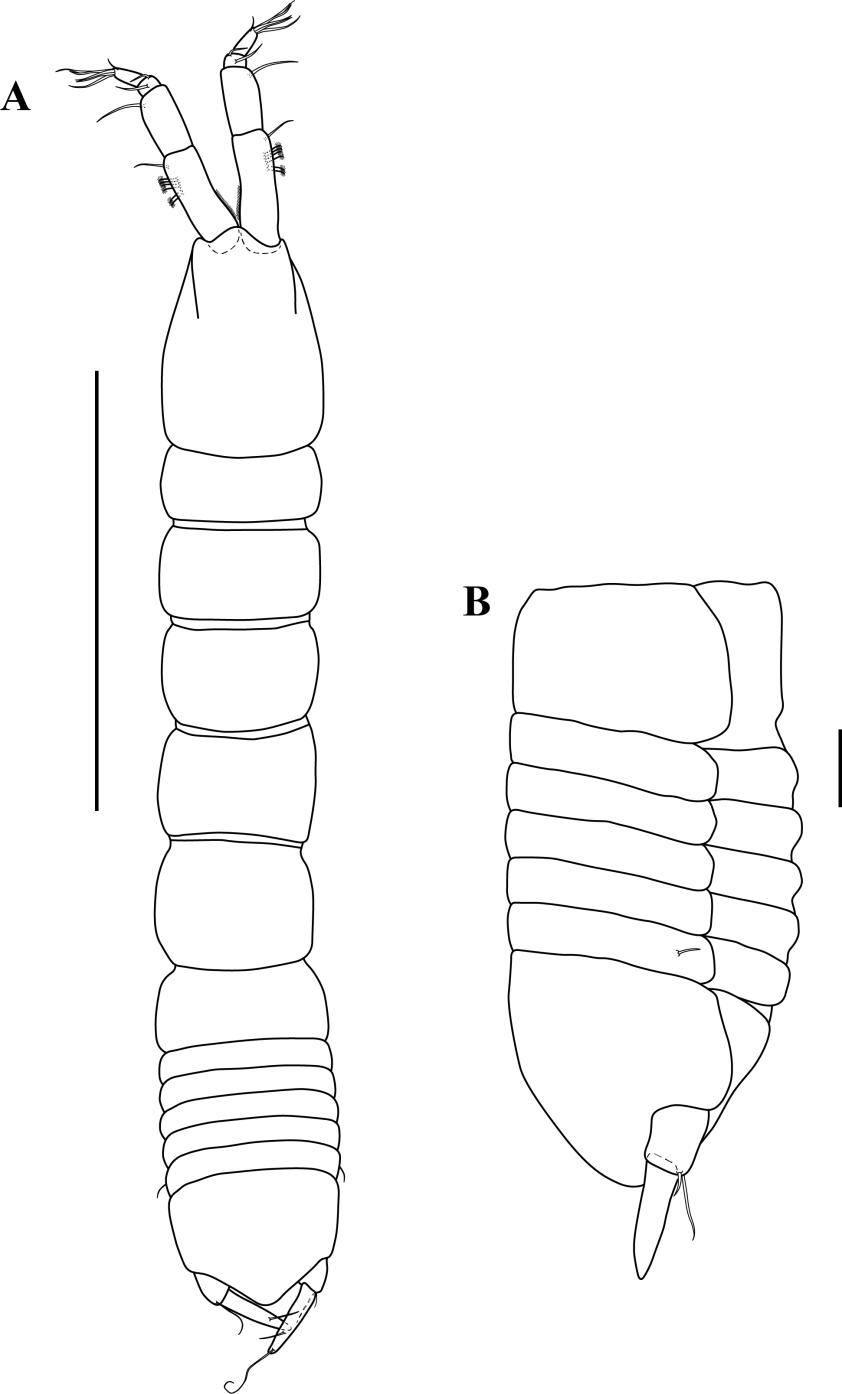
Habitus illustration. *Tanaella quintanai* sp. nov. paratype non-ovigerous female. (A) Dorsal view; (B) enlargement of pleon and pleotelson, lateral view. Scale bars = 1.0 mm for A and 0.1 mm for B.

**Figure 3:**
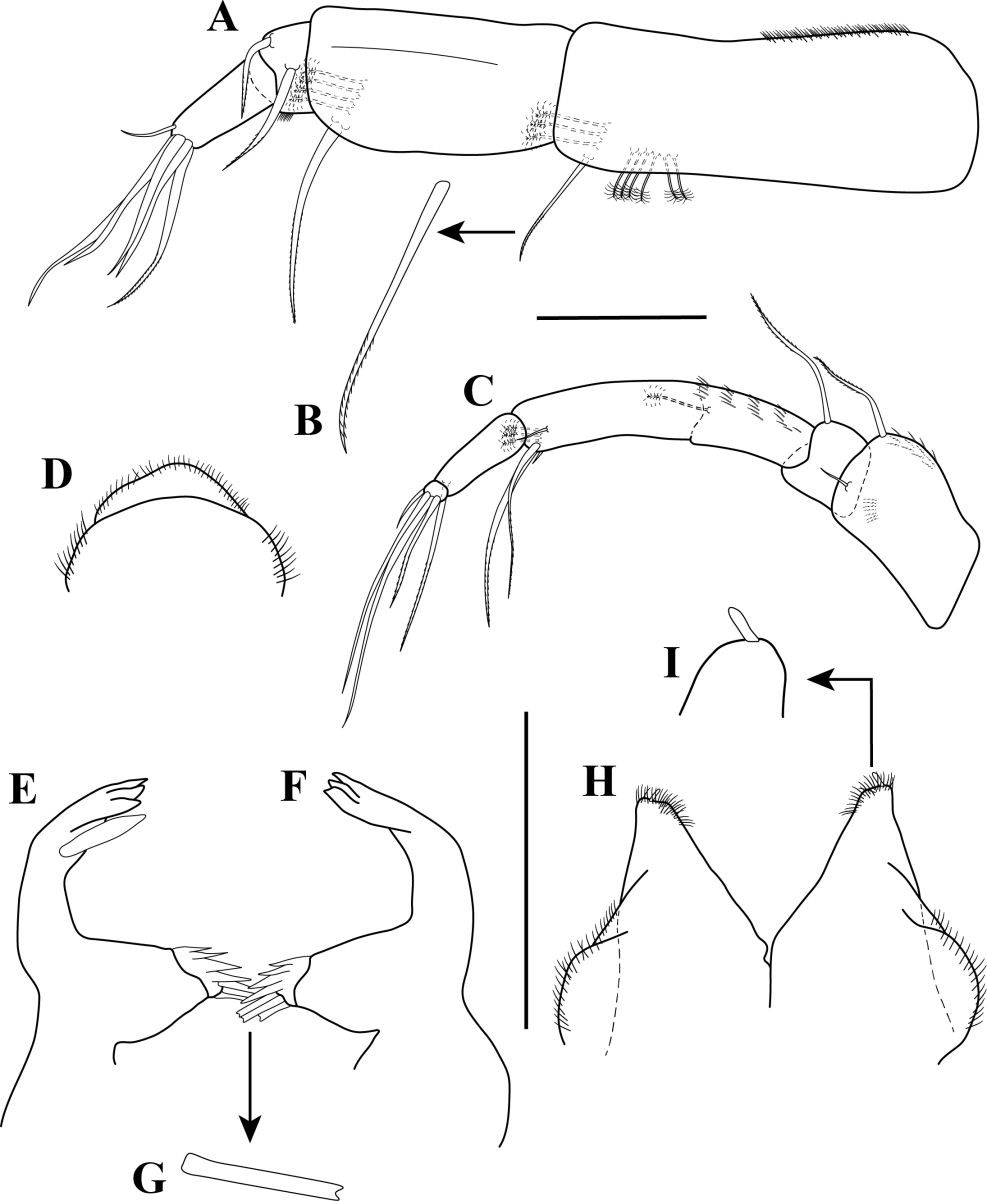
Antennule, antenna, and mouth parts illustrations. *Tanaella quintanai* sp. nov. paratype non-ovigerous female. (A) Antennule, outer view; (B) enlargement of very finely bipinnate seta; (C) antenna, outer view; (D) labrum; (E) left mandible; (F) right mandible; (G) enlargement of rectangular bifurcate seta; (H) labium; (I) enlargement of distal tip of labium palp. Scale bars = 0.1 mm for A, C–F, H.

**Figure 4:**
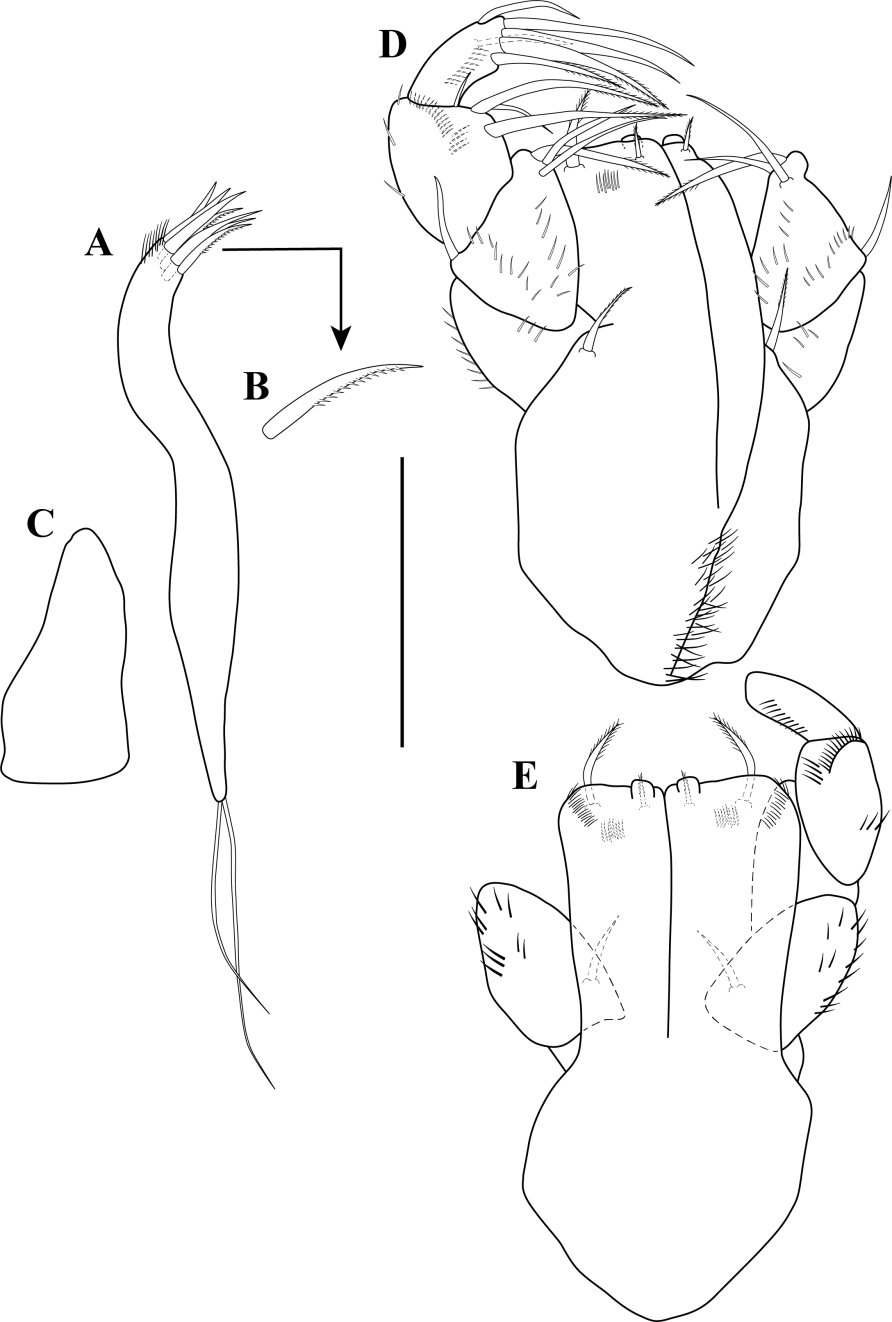
Mouth parts illustrations. *Tanaella quintanai* sp. nov. paratype non-ovigerous female. (A) Maxillule; (B) enlargement of pectinate seta; (C) maxila; (D) maxilliped; (E) endite. Scale bar = 0.1 mm for A, C–E.

**Figure 5:**
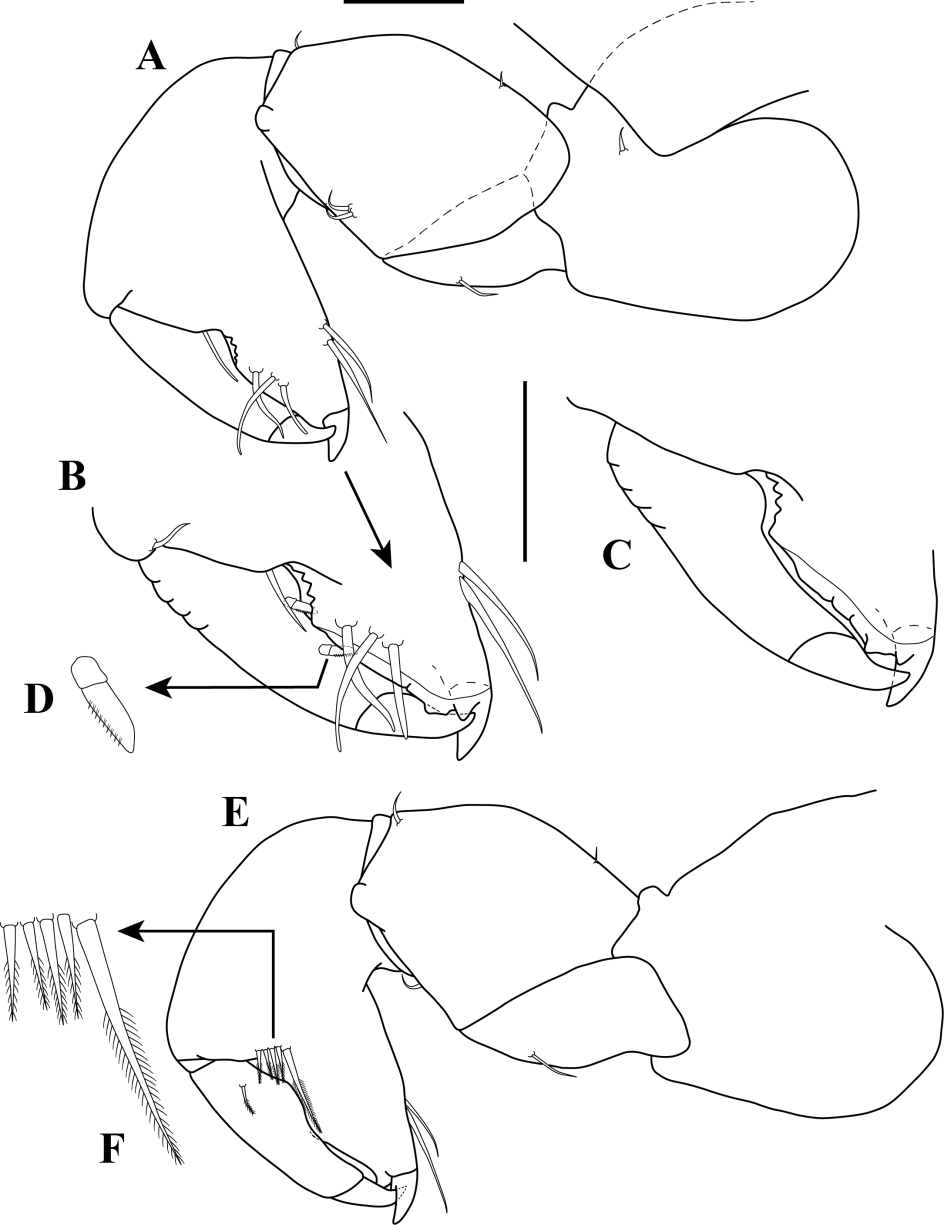
Chelipeds illustrations. *Tanaella quintanai* sp. nov. paratype non-ovigerous female. (A) Cheliped, outer view; (B) enlargement of fixed finger and dactylus; (C) enlargement of fixed finger and dactylus, setae omitted; (D) enlargement of blade-like pectinate seta; (E) cheliped, inner view; (F) enlargement of bipinnate setae. Scale bars = 0.1 mm for A–C, E.

**Figure 6:**
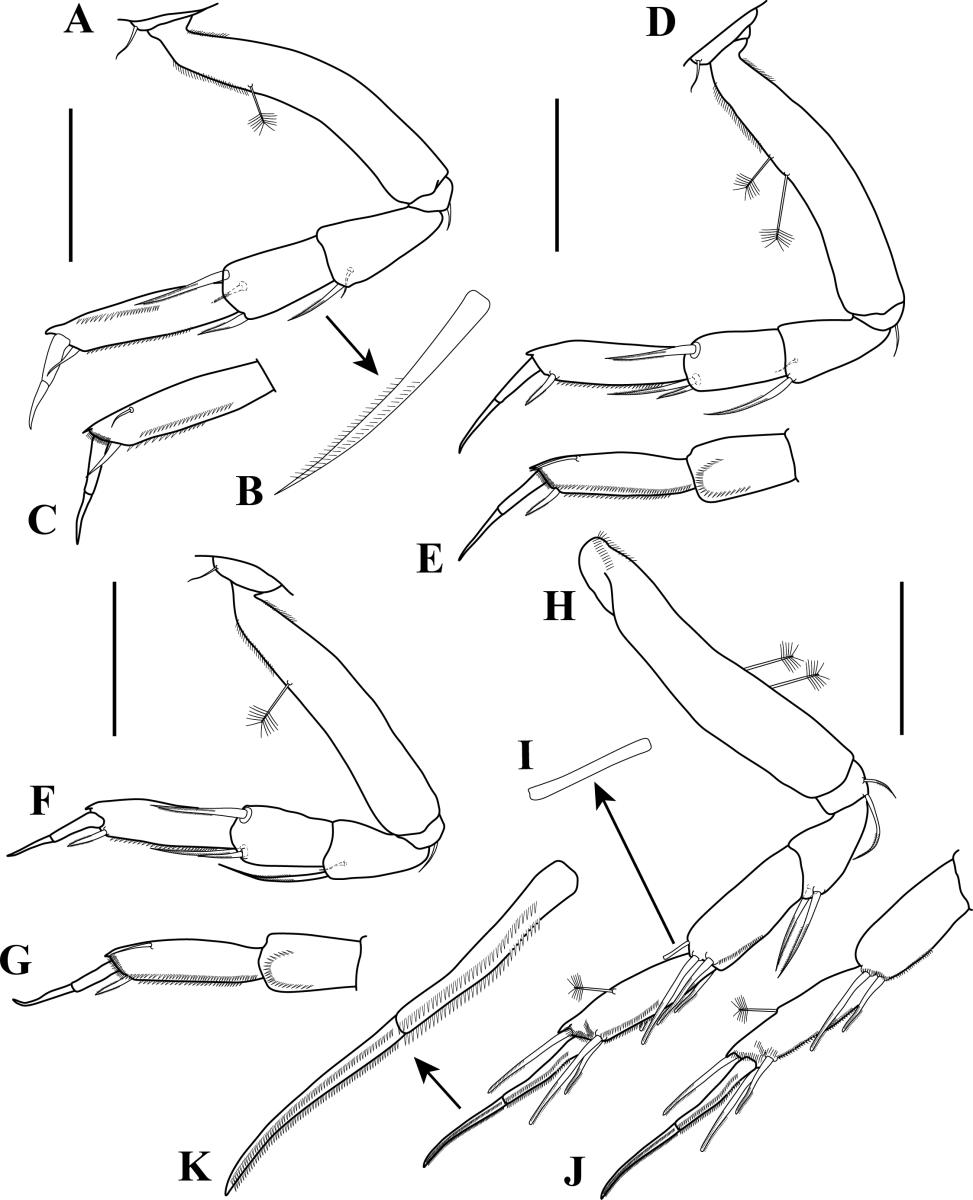
Pereopods 1–4 illustrations. *Tanaella quintanai* sp. nov. paratype non-ovigerous female. (A) Pereopod-1, outer view; (B) enlargement of bipinnate seta; (C) propodus to dactylus of pereopod-1, inner view; (D) pereopod-2, outer view; (E) carpus to dactylus of pereopod-2, inner view; (F) pereopod-3, outer view; (G) carpus to dactylus of pereopod-3, inner view; (H) pereopod-4, outer view; (I) enlargement of sub-rectangular slightly bifid bipinnate seta; (J) carpus to dactylus of pereopod-4, inner view; (K) enlargement of dactylus and unguis of pereopod-4. Scale bars = 0.1 mm for A, C–H, J.

**Figure 7:**
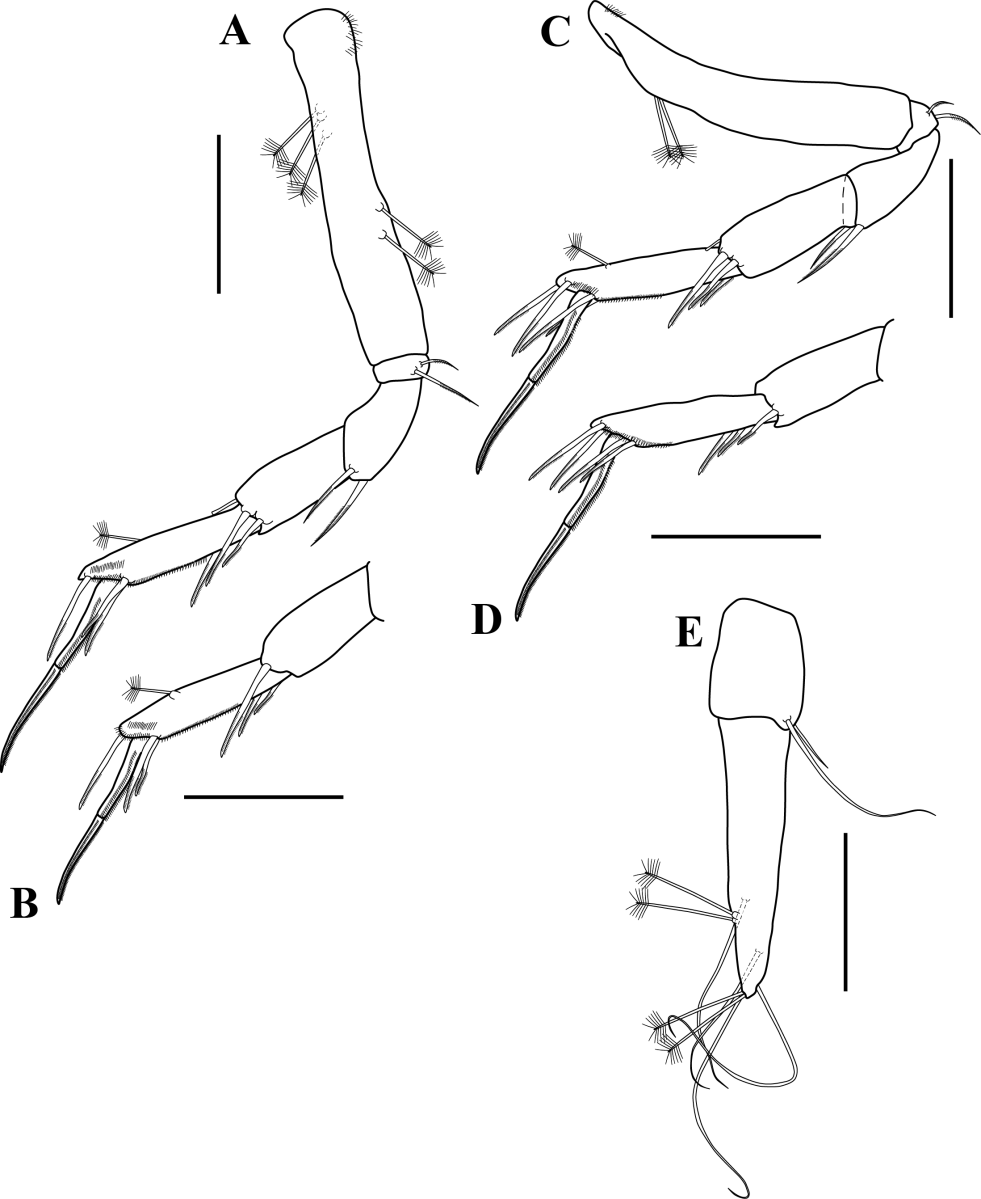
Pereopod 5–6 and uropod illustrations. *Tanaella quintanai* sp. nov., paratype non-ovigerous female. (A) Pereopod-5, outer view; (B) carpus to dactylus of pereopod-5, inner view; (C) pereopod-6, outer view; (D) carpus to dactylus of pereopod-6, inner view; (E) uropod. Scale bars = 0.1 mm for A–E.

**Figure 8:**
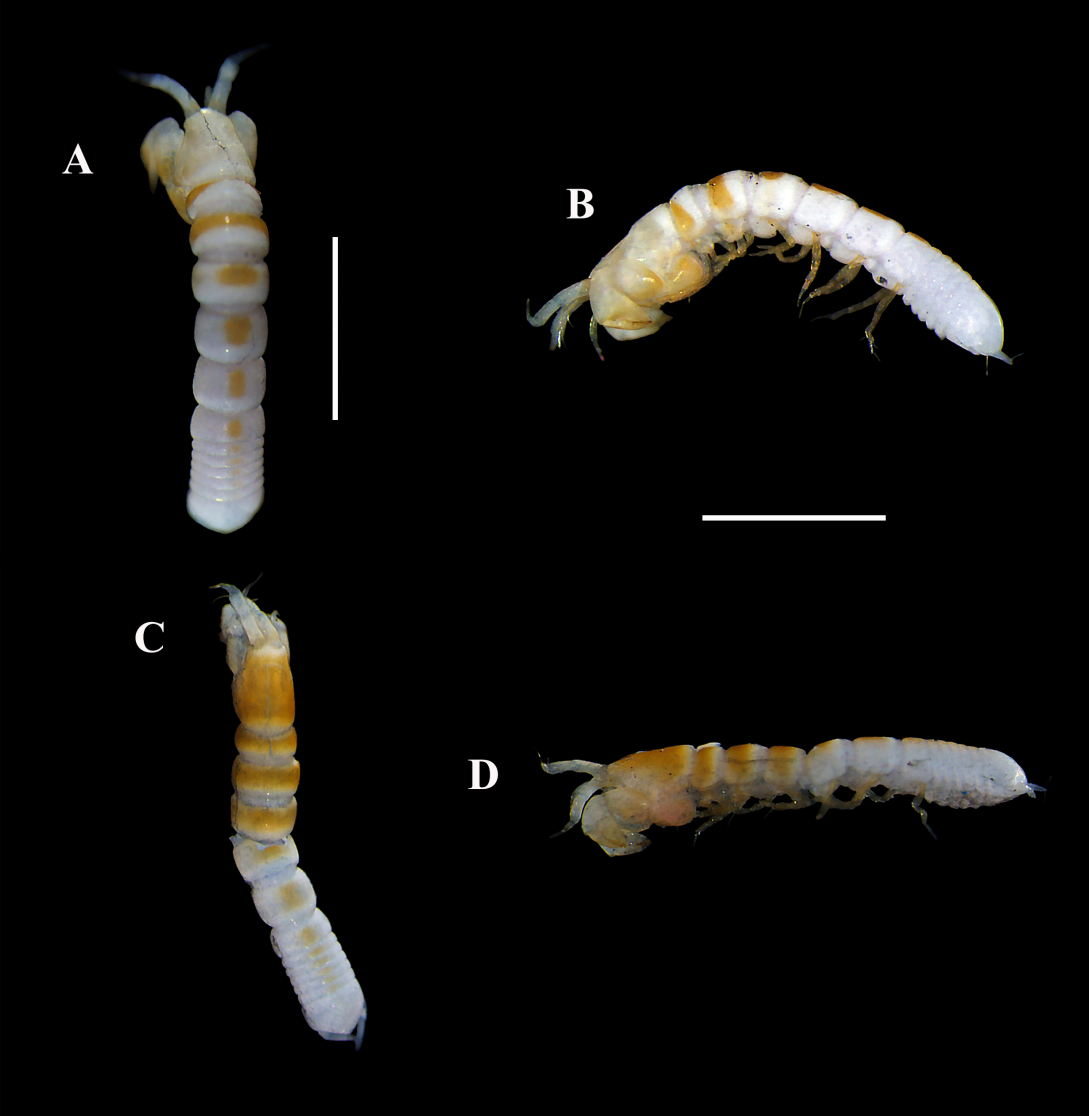
Pictures of habitus of *Tanaella quintanai* sp. nov. from Colombia. Non-ovigerous ♀, holotype (CBUMAG:MAC:01679): (A) dorsal view, TL 2.6 mm; (B) lateral view. Non-ovigerous ♀, paratype (CBUMAG:MAC:01681): (C) dorsal view, TL 2.3 mm; (D) lateral view. Scale bars = 1.0 mm. Photos by AG Morales-Núñez.

**Figure 9:**
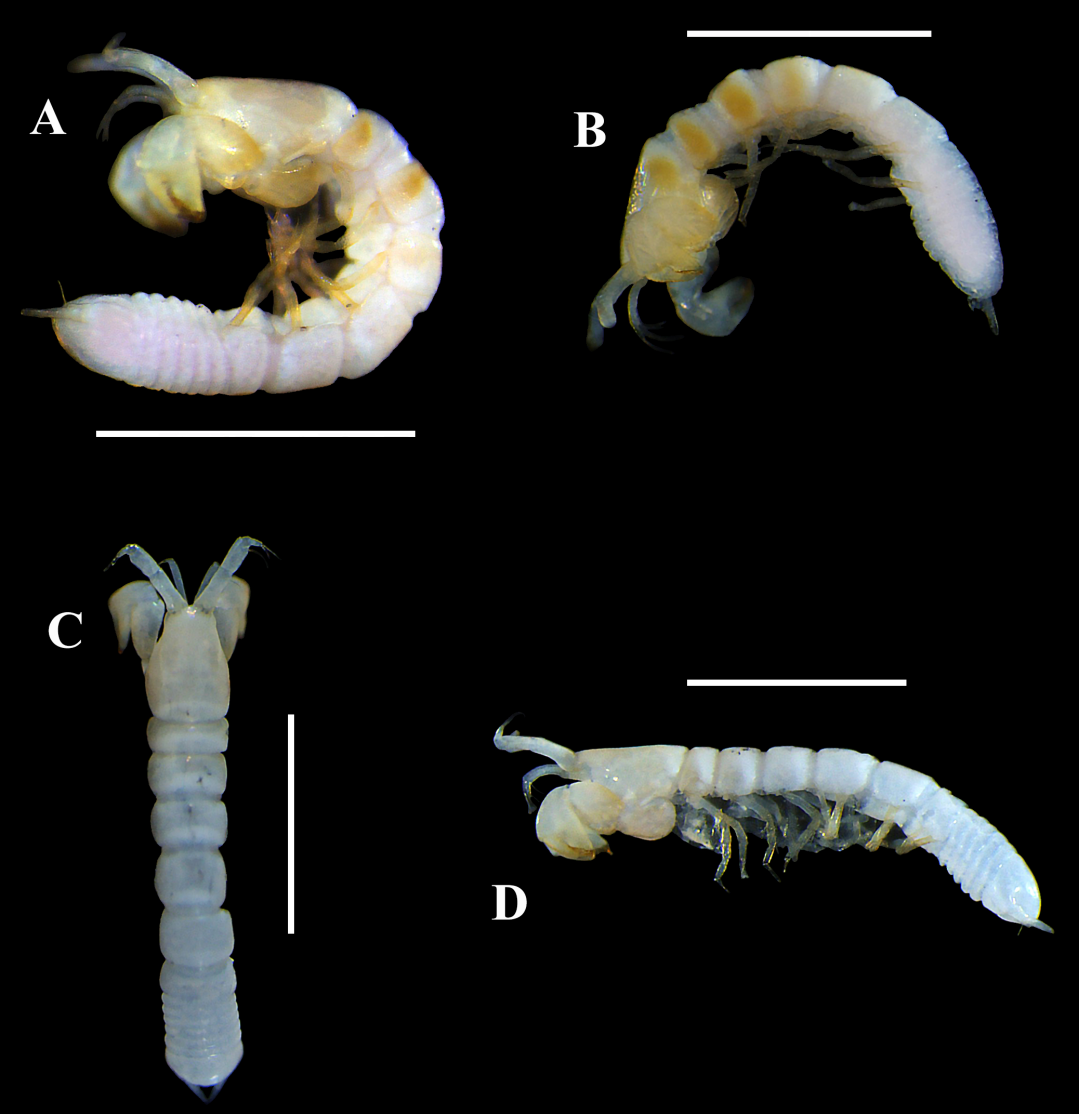
Pictures of habitus of *Tanaella quintanai* sp. nov. from Colombia. Non-ovigerous ♀, paratype (CBUMAG:MAC:01680): (A) lateral view, TL 2.2 mm. Non-ovigerous ♀, paratype (CBUMAG:MAC:01683): (B) lateral view, TL 2.4 mm. ♀ with marsupium, paratype (CBUMAG:MAC:01682): (C) dorsal view, TL 2.2 mm; (D) lateral view. Scale bars = 1.0 mm. Photos by AG Morales-Núñez.

*Paratypes*—Non-ovigerous ♀ (dissected) (CBUMAG:MAC:01681), TL 2.3 mm, Stn E15PAC (10°22′0.2″N–76°27′5.17″W), depth 2,821 m, substrata: “muddy bottom”, 26-July-2014.—Non-ovigerous ♀ (CBUMAG:MAC:01680), TL 2.2 mm, Stn E04FND (10°26′55.54″N–76°15′25.29″W), depth 2,258 m, substrata: “muddy bottom”, 09-October-2015.—Non-ovigerous ♀ (damaged) (CBUMAG:MAC:01683), TL 2.4 mm, Stn E06FSD (9°10′1.14″N–76°50′10.18″W), depth 1,659 m, substrata: “muddy bottom”, 25-April-2015. —♀ with marsupium (CBUMAG:MAC:01682), TL 2.2 mm, Stn E13PAC (10°08′59.42″N–76°32′48.77″W), depth 2,423 m, substrata: “muddy bottom”, 26-July-2014.

**Diagnosis.** *Female. Pleotelson* as long as pleonites 1–5 combined. *Antenna* article-3 with fusion line. *Labium* with apical lobe bearing one blunt seta. *Cheliped* dactylus without process and with sub-proximal bipinnate seta on inner face. *Pereopods* 1–3 with basis having sub-dorsoproximal and sub-ventroproximal margins setulose. *Pereopods 4–6* with basis having ventroproximal margin setulose and unguis bearing two parallel rows of small setules. *Pleopods* absent. *Uropod* shorter than pleotelson, basal article less than half length of endopod.

**Etymology.** Named in honor of renowned Colombian racing cyclist Nairo Alexander Quintana Rojas, in recognition and appreciation for his outstanding effort and dedication, as well as for bringing pride and happiness to the Colombian nation.

**Type locality.** Offshore waters of Córdoba department (9°14′3.8″N–76°47′14.57″W), Colombia, South America.

**Distribution.** Colombian Caribbean at depths ranging from 1,598 to 2,821 m.

**Description.** Based on non-ovigerous ♀.

*Body* (Fig. 2A). Fairly slender, TL 2.3 mm, about 6.6 times as long as wide.

*Cephalothorax* (Fig. 2A). About 21% of TL, shorter than combined lengths of pereonites 1–3, 1.4 times longer than wide, oval shape, asetose; anterior margin with rounded rostrum.

*Pereon* (Fig. 2A). About 54% of TL, all pereonites wider than long; pereonites 1 and 6 shorter than other pereonites; pereonites 2–5 increasing in length, being pereonite-5 the longest.

*Pleonites* (Figs. 2A–2B). About 12% of TL, combined lengths of pleonites 1–5 about same length that of pereonite-5, all pleonites sub-equal in length, wider than long, lacking pleopods; all pleonites laterally rounded (Fig. 2B); pleonite-5 with one pair of small lateral setae.

*Pleotelson* (Figs. 2A–2B). About 13% of TL, sub-equal length that of pleonites 1–5 combined, asetose; apex rounded.

*Antennule* (Figs. 3A–3B). Slightly shorter than cephalothorax, with four articles. Article-1 longer than combined length of articles 2–4, about three times as long as wide; ventral margin with sub-distal very finely bipinnate seta (Fig. 3B); dorsal proximal margin with setules; inner ventral margin with two (one horizontal and one oblique) sub-distal rows (six and three, respectively) of PSS. Article-2 about 1.8 times as long as wide; ventral margin with sub-distal long very finely bipinnate seta; inner ventral margin with sub-distal oblique row of four PSS. Article-3 about five times wider than long; distoventral margin setulose; mid margin with very finely bipinnate seta; sub-distal dorsal margin with very finely bipinnate seta. Article-4 length almost half of article-2, about 2.5 times as long as wide; distal margin with five (i.e., four simple, possibly very finely bipinnate, and one very finely bipinnate) setae of unequal lengths; sub-distal dorsal margin with simple seta.

*Antenna* (Fig. 3C). With five articles (article-3 with fusion line). Article-1 broader than following articles; outer mid distal margin with simple seta; anterior dorsal half margin with several rows of setules and distodorsal very finely bipinnate seta; inner mid ventral margin with oblique of setules. Article-2 shorter than article-1, sub-quadrate, with distodorsal very finely bipinnate seta. Article-3 longer than other articles, with clear fusion line; distoventral margin with one small simple seta and two long very finely bipinnate setae; mid sub-distal outer margin with simple seta; posterior dorsal half margin with several rows of setules; inner margin with one mid PSS and distal oblique row of three PSS. Article-4 longer than article-2, about 2.4 times as long as wide; inner distal margin with mid long very finely bipinnate seta. Article-5 minute, with distal margin bearing four (i.e., three simple (possibly very finely bipinnate) and one very finely bipinnate) setae of unequal lengths.

*Mouthparts*: *Labrum* (Fig. 3D) hood-shape and finely setose. *Mandibles* (Figs. 3E–3G): left mandible, with incisor with three uneven denticles, *lacinia mobilis* long, narrow (Fig. 3E). Right mandible incisor with three uneven denticles (Fig. 3F). Molar process of left and right mandibles similar, broad and blunt, with four apical denticles and three rectangular bifurcated seta (Fig. 3G).

*Labium* (Figs. 3H–3I). Two triangular lobes, each lobe with inner and distal margin setulose, apical lobe with one blunt seta (Fig. 3I).

*Maxillule* (Figs. 4A–4B). Endite with seven distal spiniform setae (two pectinate; Fig. 4B), outer distal margin with several setules; palp bearing two long terminal setae of unequal lengths.

*Maxilla* (Fig. 4C). Elongate subovate.

*Maxilliped* (Figs. 4D–4E). Basis fused, setose, each with very finely bipinnate seta near palp insertion. Endites with distal process and two pairs of very finely bipinnate setae of unequal lengths; outer margin with oblique row of setules (Fig. 4E). Palp article-1 with several rows of setules; article-2 with several rows of setules, inner distal margin with two simple setae and one very finely bipinnate seta (Fig. 4D), outer margin with simple seta; article-3 with inner margin having four setae (two very finely bipinnate and two simple) of unequal lengths (Fig. 4D), mid margin with oblique row of setules, outer margin with several rows of setulate; article-4 with distal margin bearing four setae (two very finely bipinnate and two simple) (Fig. 4D), mid margin with sub-distal simple seta and oblique row of setules (Fig. 4D), outer margin with sub-distal simple seta (Fig. 4D).

*Epignath*. Not recovered.

*Cheliped* (Figs. 5A–5F). Basis length subequal to carpus, about 1.7 times as long as wide, posterior lobe larger than anterior mass, with sub-distal dorsal seta on outer margin (Fig. 5A). Merus triangular with simple seta on mid ventral margin. Carpus about 1.4 times as long as wide; mid ventral margin with two simple setae; dorsal margin with two (one proximal and one distal) small simple setae. Propodus as long as wide, with distodorsal simple seta (Fig. 5B), palm 1.4 times longer than fixed finger, outer margin with simple seta near to insertion of dactylus, inner face with five bipinnate setae (one distinctly longest) near to articulation of dactylus (Figs. 5E–5F); fixed finger with two ventral setae, with three sub-marginal simple setae on outer incisive margin, cutting edge with proximal serrated depression (Figs. 5A–5C) and five (three rounded and two sharp) denticles (Fig. 5C), claw short. Dactylus and unguis curving downward; ventral margin with two small blade-like pectinate setae (Fig. 5D); inner face with bipinnate seta on sub-proximal margin (Fig. 5E).

*Pereopod*-*1* (Figs. 6A–6C). Coxa with simple seta on anterodistal margin. Basis as long as combined length of ischium, merus, carpus, and half of propodus, about six times as long as wide; sub-ventroproximal margin setulose; sub-dorsoproximal margin setulose with one PSS. Ischium wider than long, with one simple seta on ventral margin. Merus slightly shorter than carpus, about 2.2 time as long as wide; distoventral margin with bipinnate seta (Fig. 6B); inner margin with sub-distal simple seta. Carpus about two times as long as wide; distoventral margin with bipinnate seta; distodorsal margin with bipinnate seta, inner margin with mid sub-distal bipinnate seta. Propodus about four times as long as wide; ventral margin with two (one on inner view (Fig. 6C)) rows of spinules and one distal spiniform seta (possibly bipinnate); dorsal margin with a row of setules (Fig. 6A) and distally with spine-like apophysis; inner face with sub-distal dorsal simple seta and distally setose (Fig. 6C). Dactylus together with unguis shorter than propodus; dactylus slightly longer than unguis.

*Pereopod-2* (Figs. 6D–6E). Similar to pereopod-1 except: shorter; basis with two PSS on dorsal margin; merus with inner face having a semi-circle of setules (Fig. 6E); carpus with distoventral margin with two bipinnate setae; propodus with distoventral bipinnate seta; dactylus slightly shorter than unguis.

*Pereopod-3* (Figs. 6F–6G). Similar to peropod-2 except: shorter; basis with one PSS on dorsal margin; merus shorter than carpus; dactylus slightly longer than unguis

*Pereopod-4* (Figs. 6H–6K). Without coxa. Basis slightly wider than on pereopod 1–3, about five times as long as wide; ventroproximal margin setulose, with two PPS on mid-ventral margin. Ischium wider than long, with two bipinnate setae of unequal lengths on ventral margin. Merus shorter than carpus, about 1.9 times as long as wide, with two distoventral bipinnate setae. Carpus length subequal to propodus, about 2.5 times as long as wide; distoventral half margin setulose, with two mid-distal bipinnate setae; distodorsal margin with one sub-rectangular slightly bifid bipinnate seta (Fig. 6I); inner face with two mid-distally bipinnate setae (Fig. 6J). Propodus three times as long as wide; ventral margin with two rows of setules, distally with two bipinnate setae; mid-outer sub-distal and distal margin setose; dorsal margin with mid PSS, distally with spine-like apophysis and one bipinnate seta; mid-inner face sub-distal and distal margin setose (Fig. 6J). Dactylus together with unguis longer than propodus, dactylus length subequal to unguis, dactylus and unguis with two parallel rows of small setules (Fig. 6K).

*Pereopod-5* (Figs. 7A–7B). Similar to peropod-4 except: slightly shorter; basis with three PSS on dorsal margin; inner face of carpus with one mid-distally bipinnate seta (Fig. 7B); dactylus shorter than unguis.

*Pereopod-6* (Figs. 7C–7D). Similar to pereopod-5 except: slightly longer; basis with two PSS on dorsal margin; propodus with two bipinnate setae on distodorsal margin.

*Pleopods*. Absent.

*Uropod* (Figs. 2B–7E). Shorter than pleotelson (Fig. 2B). Basal article less than half length of endopod, about 1.3 times as long as wide, with two (one long and one short) distal simple setae on outer margin. Exopod vestigial, indicated by two simple setae. Endopod one-articled, about five times as long as wide, with four (two sub-distal and two distal) PSS, with two sub-distal and two distal simple setae of unequal lengths.

Male. Unknown.

### Size-distribution

The body sizes of *Tanaella quintanai* individuals were measured and presented in Table 1. The non-ovigerous females ranged from 2.2 mm to 2.6 mm (*n* = 4) (Fig. 8, 9A–9B). Female with marsupium TL 2.2. mm (Figs. 9C–9D)

### Color in alcohol

Upon being preserved in 70% ethanol for more than four years, the four non-ovigerous females (Figs. 8, 9A–9B) presented a yellowish-orange coloration (intensity can vary) on some parts of the habitus and appendages as follows: (1) carapace yellowish-orange (intensity can vary), (2) pereonites 1–2 (Figs. 8A–8B) or 1–3 (Figs. 8C–8D, 9A–9B) with dorso transverse yellowish-orange band, (3) pereonites 3–6 (Figs. 8A–8B) or 4–6 (Figs. 8C–8D) and pleonites 1–5 with mid-dorsal yellowish-orange spot, the size and color intensity of spots decreased from pereonite 3–4 to pleonite-5, (4) cheliped yellowish-orange, and (5) all pereopods yellowish-orange. The only female examined specimen that had a marsupium, presented a weaker yellowish-orange coloration of the habitus and appendages than the non-ovigerous females (Figs. 8B, 8D,9A–9B).

### Ecological notes

Specimens of *T. quintanai* **sp. nov.** (Figs. 8 and 9) were collected from muddy bottoms with a content of mud and clay between 94 and 98%. Other physicochemical parameters of the surrounding waters were a temperature of 4.1–4.4 °C, salinity of 35 ppm, pH of 7.7–8.0, and DO of 4.7–6.8 mg/L.

## Discussion

**Figure 10:**
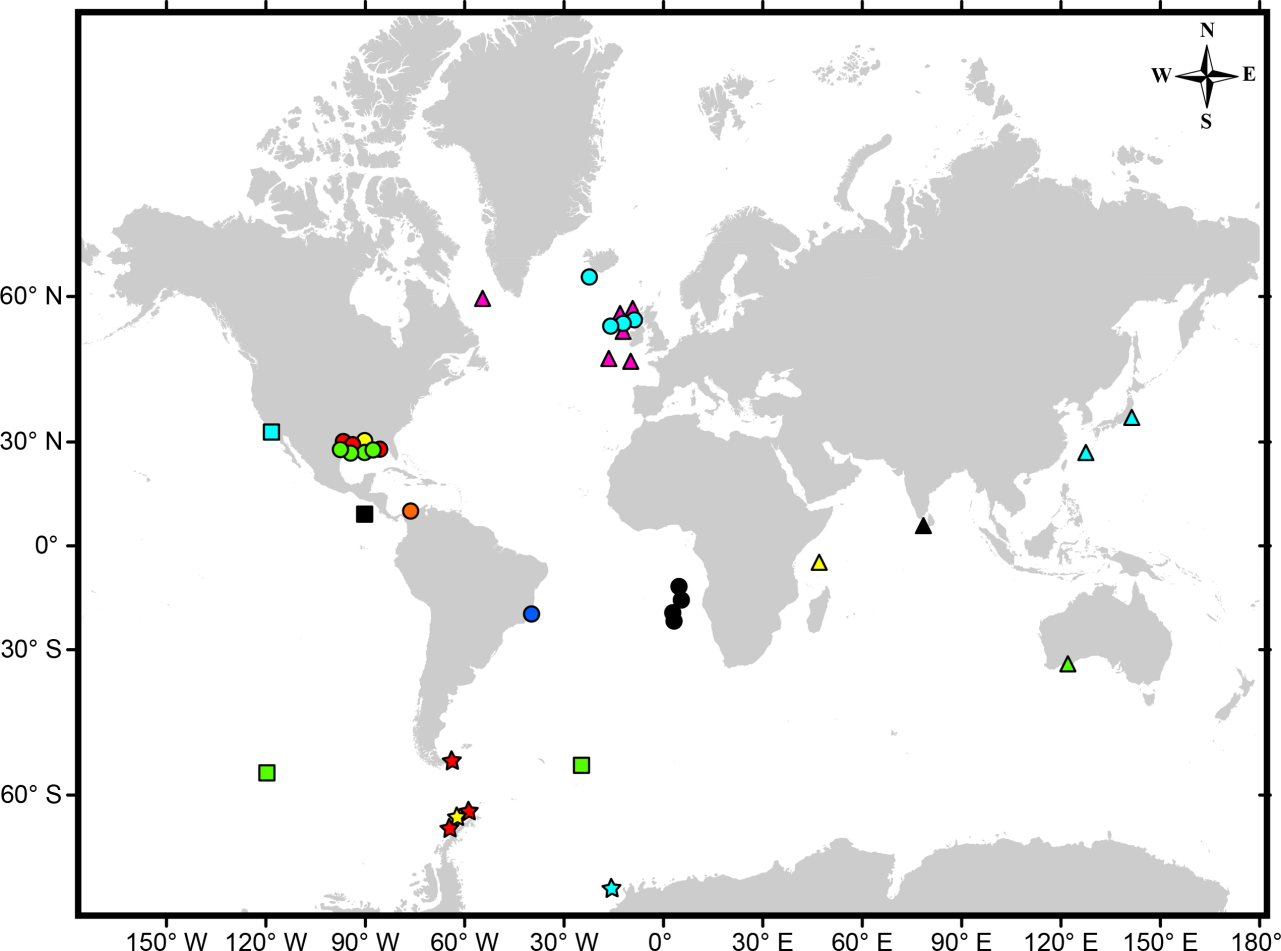
Map showing the worldwide distribution of *Tanaella*. *T. busteri* (yellow circles) (Drumm & Bird, 2016), *T. dongo* (light green triangle) (Bamber, 2005), *T. eltaninae* (light green squares) (Guerrero-Kommritz & Błazewicz-Paszkowycz, 2004), *T. forcifera* (black square) (Lang, 1968; Larsen & Heard, 2004), *T. kimi* (cyan star) (Guerrero-Kommritz & Błazewicz-Paszkowycz, 2004), *T. kommritzia* (cyan triangles) (Larsen & Shimomura, 2007), *T. kroyeri* (blue circle) (Larsen, Araújo-Silva & Coelho, 2009), *T. mclellandi* (red circles) (Larsen & Heard, 2004), *T. ochracea* (magenta triangles) (Hansen, 1913; Larsen & Heard, 2004), *T. paraforcifera* (yellow triangle) (Lang, 1968; Larsen & Heard, 2004), *T. profunda* (black circles) (Guerrero-Kommritz & Błazewicz-Paszkowycz, 2004), *T. prolixcauda* (light green circles) (Larsen & Heard, 2004), *T. propinquus* (cyan square) (Dojiri & Sieg, 1997; Larsen & Heard, 2004), *T. quintanai* sp. nov. (orange circle), *T. rotundicephala* (yellow star) (Sieg, 1986; Larsen & Heard, 2004), *T. tuberculata* (black triangle) (Kudinova-Pasternak, 1989; Larsen & Heard, 2004), *T. unguicillata* (cyan circles) (Norman & Stebbing, 1886; Larsen & Heard, 2004), *T. unisetosa* (red stars) (Sieg, 1986; Larsen & Heard, 2004).

With the description of new Colombian species, seven of 18 species of *Tanaella* (Table 2) are known to be from the western Atlantic Ocean; among them, *Tanaella ochracea* from the western tip of Greenland, three (*T. busteri*, *T. mclellandi*, and *T. prolixcauda*) from Gulf of Mexico, *T. kroyeri* from off Brazil, *T. unisetosa* from the tip of Argentina, and *T. quintanai* **sp. nov.**, which represents the first species of the genus described from the Caribbean (Fig. 10). During an earlier study by Morales-Núñez & Ardila (2018), it was first reported as *Tanaella* sp. in Caribbean waters, establishing the presence of the Family Tanaellidae in deep-waters off the Colombian Coast.

**Table 2:** Recognized species of the genus *Tanaella*. Alphabetical listing of the 18 currently recognized species of the genus *Tanaella*, including information on distribution and depth range.

**Species**	**Geographical area**	**Depth range (m)**
*T. busteri*Drumm & Bird, 2016^a^	North western Atlantic Ocean (Gulf of Mexico)	2,221–2,289
*T. dongo*Bamber, 2005	South eastern Indian Ocean (Australia)	38.8
*T. eltaninae*Guerrero-Kommritz & Błazewicz-Paszkowycz, 2004	Antarctica	3,978–4,742
*T. forcifera* (Lang, 1968)	North eastern Pacific Ocean (Mexico-Panama)	3,570
*T. kimi*Guerrero-Kommritz & Błazewicz-Paszkowycz, 2004^a^	Antarctica	2,077
*T. kommritzia*Larsen & Shimomura, 2007	North western Pacific Ocean (Japan)	169–654
*T. kroyeri*Larsen, Araújo-Silva & Coelho, 2009^a^	South western Atlantic Ocean (Brazil)	650–1,480
*T. mclellandi*Larsen & Heard, 2004^a^	North western Atlantic Ocean (Gulf of Mexico)	213–2,920
*T. ochracea*Hansen, 1913^a^	North western (tip of Greenland) and eastern Atlantic Ocean	1,200–4,834
*T. paraforcifera* (Lang, 1968)^a^	South western Indian Ocean (Madagascar-Mombasa)	4,800
*T. profunda*Guerrero-Kommritz & Błazewicz-Paszkowycz, 2004^a^	South eastern Atlantic Ocean (Angola)	5,180–5,447
*T. prolixcauda*Larsen & Heard, 2004^a^	North western Atlantic Ocean (Gulf of Mexico)	550–2,030
*T. propinquus*Dojiri & Sieg, 1997	North eastern Pacific Ocean (Mexico)	59–320
*T. quintanai***sp. nov.**	North western Atlantic Ocean (Caribbean)	1,598–2,821
*T. rotundicephala*Sieg, 1986	Antarctica	119–124
*T. tuberculata*Kudinova-Pasternak, 1989^a^	North Indian Ocean	3,660
*T. unguicillata*Norman & Stebbing, 1886 [**Type species]^a^**	North eastern Atlantic Ocean and Mediterranean Sea	176–2,900
*T. unisetosa*Sieg, 1986	North western Atlantic (tip of Argentina) and Antarctica	44–137

**Notes.**

a Indicates male known.

Sixteen out of eighteen of the described species of *Tanaella* have included at least a female, with only two species been described as males: *T. busteri* and *T. prolixcauda*. The new species from the Caribbean differs from *T. busteri* and *T. prolixcauda* by having (1) labium with apical lobe having a blunt seta (absent in *busteri* and *prolixcauda*) (Table 3), (2) cheliped with dactylus having a sub-proximal bipinnate seta on inner face (absent in *busteri* and *prolixcauda*) (Table 3), (3) pereopods 1–3 with basis having sub-dorsoproximal and sub-ventroproximal margins setulose (lacking setulose margins in *busteri* and *prolixcauda*) (Table 3), (4) pleotelson as long as pleonites 1–5 combined (shorter in *busteri* and *prolixcauda*) (Tables 1 and 3), and (5) uropod shorter than pleotelson (longer in *busteri* and *prolixcauda*) (Table 3).

*Tanaella quintanai* can be differentiated from *T*. *ochracea* and *T. unisetos* a by (1) cheliped with dactylus having a sub-proximal bipinnate seta on inner face (absent in *ochracea* and *unisetosa*) (Table 3), (2) pereopods 1–3 with basis having sub-dorsoproximal and sub-ventroproximal margins setulose (lacking setulose margins in *ochracea* and *unisetosa*) (Table 3), (3) pereopods 4–6 with basis having ventroproximal margin setulose (lacking setulose margin in *ochracea* and *unisetosa*) (Table 3), (4) pereopods 4–6 with unguis bearing two parallel rows of small setules (lacking parallel rows of small setules in *ochracea* and *unisetosa*), (5) pleotelson as long as pleonites 1–5 combined (shorter in *ochracea* and *unisetosa*) (Tables 1 and 3), and (6) uropod shorter than pleotelson (longer in *ochracea* and *unisetosa*).

*Tanaella quintanai* closely resembles *T*. *kroyeri* and *T. mclellandi* by having antennal article-3 with clear fusion line and uropod shorter than pleotelson (Table 3). *Tanaella quintanai*; however, can be distinguished from *T. kroyeri* and *T*. *mclellandi* by: (1) cheliped with dactylus having a sub-proximal bipinnate seta on inner face (absent in *kroyeri* and *mclellandi*) (Table 3), (2) pereopods 1–3 with basis having sub-dorsoproximal and sub-ventroproximal margins setulose (lacking setulose margins in *kroyeri* and *mclellandi*) (Table 3), (3) pereopods 4–6 with basis having ventroproximal margin setulose (lacking setulose margin in *kroyeri* and *mclellandi*) (Table 3), (4) pereopods 4–6 with unguis bearing two parallel rows of small setules (lacking parallel rows of small setules in *kroyeri* and *mclellandi*), and (5) pleotelson as long as pleonites 1–5 combined (shorter in *kroyeri* and *mclellandi*) (Tables 1 and 3).

Aside from the features indicated above, which were utilized to separate *Tanaella quintanai* from his congeners, *Tanaella quintanai* appears to be similar to *T. unisetosa* by having a maxilliped basis setose (Table 3), and also seems to be similar to *T*. *forcifera* by having the cheliped basis with sub-distal simple seta on dorsal margin (Table 3). Although it might be possible that these two characters have been overlooked and were not mentioned on the original descriptions and re-descriptions (Larsen & Heard, 2004) along the other members (i.e., 15 species) of the genus *Tanaella*. This indicates examination of addition type material or topotypic specimens of the other members of the genus needed to determine whether or not they exhibit these features.

**Table 3:** Variant characters among *Tanaella* species. A comparison of some variant characters among *Tanaella* species. Data from: Drumm & Bird (2016), Bamber (2005), Guerrero-Kommritz & Błazewicz-Paszkowycz (2004), Lang (1968), Larsen & Shimomura (2007), Larsen, Araújo-Silva & Coelho (2009), Larsen & Heard (2004), Hansen (1913), Dojiri & Sieg (1997), Sieg (1986), Kudinova-Pasternak (1989) and Norman & Stebbing (1886), this study.

**Species**	**Antenna article-3 or -4 fusion line**	**Labium with seta or spinule on apical lobe**	**Maxilliped basis setose/asetose**	**Cheliped basis with sub-distal simple seta on dorsal margin**	**Cheliped dactylus inner face with seta on sub-proximal margin**	**Pereopods 1–3 dorso-ventral proximal margin setulose**	**Pereopods 4–6 ventral proximal margin setulose**	**Pleotelson shorter/longer/as long as that length of pleon**	**Uropod shorter/longer/as long as than length of pleotelson**	**Uropod endopod articles**
*T. busteri*	Present (article-4)	Absent	Asetose	Absent	Absent	Absent	Absent	Shorter	Longer	1
*T. dongo*	Absent	Absent	Asetose	Absent	Absent	Absent	Absent	Shorter	Shorter (about half as long as pleotelson)	2
*T. eltaninae*	Absent	Spiniform	Asetose	Absent	Absent	Only P-1 (ventrally)	Absent	Shorter	Shorter (more than two thirds as long as pleotelson)	1
*T. forcifera*	Absent	Absent	Asetose	Present*	Simple	Absent	Absent	Shorter	Longer++	2
*T. kimi*	Absent	Spiniform	Asetose	Absent	Absent	Present (dorsally)	Absent	Shorter	Shorter (more than two thirds as long as pleotelson)	1
*T. kommritzia*	Present (article-4)	Absent	Asetose	Absent	Absent	Absent	Absent	Shorter	Longer++	1
*T. kroyeri*	Present (article-3)	Absent	Asetose	Absent	Absent	Absent	Absent	Shorter	Shorter (more than two thirds as long as pleotelson)	1
*T. mclellandi*	Present (article-3)	n/i	Asetose	Absent	Absent	Absent	Absent	Shorter	Shorter (about half as long as pleotelson)	1
*T. ochracea*	Present (article-3)	Absent	Asetose	Absent	Absent	Absent	Absent	Shorter	Longer	1
*T. paraforcifera*	Absent	Spinule	Asetose	Absent	Simple	Absent	Absent	Shorter	As long as	1
*T. profunda*	Absent	n/i	Asetose	Absent	Absent	Only P-1 (dorsally)	Absent	Shorter	Longer	1
*T. prolixcauda*	Present (article-3)	Absent	Asetose	Absent	Absent	Absent	Absent	Shorter	Longer	1
*T. propinquus*	n/i	n/i	n/i	Absent	Present*	Absent	Absent	Shorter	Longer	2
*T. quintanai***sp. nov**.	Present (article-3)	**Blunt**	Setose	Present	**Bipinnate**	Present	**Present**	As long as	Shorter (more than two thirds as long as pleotelson)	1
*T. rotundicephala*	Present (article-4)	Absent	Setose	Absent	Absent	Absent	Absent	Short	Longer	2
*T. tuberculata*	Absent	Absent	Asetose?	Absent	Absent	Absent	Absent	Shorter	Longer	1
*T. unguicillata*	Present (article-3)	Absent	Asetose	Absent	Absent	Present	Absent	As long as	Shorter (more than two thirds as long as pleotelson)	1
*T. unisetosa*	Absent	Absent	Setose	Absent	Absent	Absent	Absent	Shorter	Longer	2

**Notes.**

n/i not information from original description * Character not mentioned, but illustrated on the original description ++ Character mentioned, but it looks different on the original illustration

*Tanaella quintanai* can be differentiated from the other species of *Tanaella*, as shown in the following identification key and Table 3.

### Key to the known species (females) of *Tanaella*, modified from Drumm & Bird, 2016

**Table:** 

1. Uropod endopod two-articled …**2**
– Uropod endopod one-articled …**6**
2. Cephalothorax as long as broad …*T. rotundicephala* (♀; ♂ unknown)
– Cephalothorax longer than broad …**3**
3. Uropods shorter than length of pleotelson …*T. dongo* (♀; ♂ unknown)
– Uropods longer than length of pleotelson …**4**
4. Pleotelson as long as combined length of first two pleonites …*T. propinquus* (♀; ♂ unknown)
– Pleotelson longer than combined length of first two pleonites …**5**
5. Antennule article-1 shorter than combined length of last three articles …*T. unisetosa* (♀; ♂ unknown)
– Antennule article-1 longer than combined length of last three articles …*T. forcifera* (♀; ♂ unknown)
6. Uropods longer than pleotelson …**7**
– Uropods as long as, or shorter than, pleotelson …**10**
7. Pleotelson apex pointed …*T. tuberculata* (♀; ♂)
– Pleotelson rounded apex …**8**
8. Uropod basal article as long as endopod …*T. kommritzia* (♀; ♂ unknown)
– Uropod basal article shorter than endopod …**9**
9. Pleotelson as long as combined length of three pleonites …*T. ochracea* (♀; ♂)
– Pleotelson as long as combined length of four pleonites …*T. profunda* (♀; ♂)
10. Cheliped dactylus with proximal process on inner margin …*T. unguicillata* (♀; ♂)
– Cheliped dactylus without proximal process on inner margin …**11**
11. Uropod as long as pleotelson …*T. paraforcifera* (♀; ♂)
– Uropod shorter than pleotelson …**12**
12. Cheliped with dactylus having a sub-proximal bipinnate seta on inner face. Pereopods 4–6 basis with ventroproximal margin setulose. Pleotelson as long as pleonites 1–5 combined … *T. quintanai***sp. nov.** (♀; ♂ unknown)
– Cheliped with dactylus lacking a sub-proximal bipinnate seta on inner face. Pereopods 4–6 basis without ventroproximal margin setulose. Pleotelson shorter than pleonites 1–5 combined …**13**
13. Pereopod-1 basis dorsal margin with minute seta …*T. kimi* (♀; ♂)
– Pereopod-1 basis dorsal margin without minute seta …**14**
14. Uropod about half as long as pleotelson …*T. mclellandi* (♀; ♂)
– Uropod more than two-thirds as long as pleotelson …**15**
15. Pereopod-1 dactylus together with unguis longer than propodus. Pereopod-1 dactylus asetose …*T. kroyeri* (♀; ♂)
– Pereopod-1 dactylus together with unguis shorter than propodus. Pereopod-1 dactylus with sub-proximal simple seta …*T. eltaninae* (♀; ♂ unknown)

## Conclusions

This is the first tanaellid species described from Colombian waters and the only member of the genus *Tanaella* reported from the Caribbean; increasing the distribution range of the genus to the southern area of the Caribbean Sea (Fig. 10). *Tanaella quintanai* can be easily distinguished from all its congeners by having pereopods 4–6 with basis bearing ventroproximal margin setulose and pereopods 4–6 with unguis bearing two parallel rows of small setules. The lack of records for *Tanaella* in the coastal and deep waters of the Caribbean may be due to the lack of sampling in live-bottom habitats.

##  Supplemental Information

10.7717/peerj.7571/supp-1 File S1 Raw data-specimens of *Tanaella quintanai* Geographical information, physicochemical data and specimens of Spyrapodids used in this study.

## Abbreviations

ANLA: National Authority of Environmental Licenses
CBUMAG: Centro de Colecciones Biológicas, Universidad del Magdalena, Santa Marta, Colombia
FSD: Fuerte Sur
FND: Fuerte Norte
PAC: Purple Angel Block
TL: Total body length
PSS: Plumose sensory setae
Stn: Station

## Additional Information and Declarations

### Field Study Permissions

The following information was supplied relating to field study approvals (i.e., approving body and any reference numbers): Collection permits were granted by the National Authority of Environmental Licenses—ANLA. The permit numbers were: FSD, ANLA No. 0723 de 2012; FND, ANLA No. 1016 de 2012; PAC, ANLA No. 0880 de 2014.

### Data Availability

The following information was supplied regarding data availability: Raw data is available as Supplemental File. *Tanaella quintanai* sp. nov. (Accession numbers) CBUMAG:MAC:01679: Female (Holotype); CBUMAG:MAC:01680: Female; CBUMAG:MAC:01681: Female (dissected); CBUMAG:MAC:01682: Female with marsupium; CBUMAG:MAC:01683: Female. Material examined. Holotype—non-ovigerous ♀ (CBUMAG:MAC:01679), TL 2.6 mm, Station (Stn) E05PAC (9°14′3.8″N–76°47′14.57″W), depth 1,598 m, substrata: “muddy bottom”, 27-July-2014. Paratypes—Non-ovigerous ♀ (dissected) (CBUMAG:MAC:01681), TL 2.3 mm, Stn E15PAC (10°22′0.2″N–76°27′5.17″W), depth 2,821 m, substrata: “muddy bottom”, 26-July-2014.—Non-ovigerous ♀ (CBUMAG:MAC:01680), TL 2.2 mm, Stn E04FND (10°26′55.54″N–76°15′25.29″W), depth 2,258 m, substrata: “muddy bottom”, 09-October-2015.—Non-ovigerous ♀ (damaged) (CBUMAG:MAC:01683), TL 2.4 mm, Stn E06FSD (9°10′1.14″N–76°50′10.18″W), depth 1,659 m, substrata: “muddy bottom”, 25-April-2015.—♀ with marsupium (CBUMAG:MAC:01682), TL 2.2 mm, Stn E13PAC (10°08′59.42″N–76°32′48.77″W), depth 2,423 m, substrata: “muddy bottom”, 26-July-2014.

### New Species Registration

The following information was supplied regarding the registration of a newly described species: Publication LSID: urn:lsid:zoobank.org:pub:BB577909-6C9F-4008-AA2D-2330525FAACE. *Tanaella quintanai* sp. nov. LSID: urn:lsid:zoobank.org:act:C9C6AD95-01BB-46F8-92D8-426916C451AD.

## References

[ref-1] Bamber RN, Wells FE, Walker DI, Kendrick GA (2005). The tanaidaceans (Arthropoda: Crustacea: Peracarida: Tanaidacea) of Esperance, Western Australia, Australia. The marine Flora and Fauna of Esperance, Western Australia.

[ref-2] Bird GJ (2011). Paratanaoidean tanaidaceans (Crustacea: Peracarida) from littoral and shallow sublittoral habitats in New Zealand, with descriptions of three new genera and seven new species. Zootaxa.

[ref-3] Dojiri M, Sieg J, Blake JA, Scott PH (1997). The Tanaidacea. Taxonomic Atlas of the Benthic Fauna of the Santa Maria Basin and Western Santa Barbara channel.

[ref-4] Drumm DT, Bird GJ (2016). New deep-sea Paratanaoidea (Crustacea: Peracarida: Tanaidacea) from the northeastern Gulf of Mexico. Zootaxa.

[ref-5] Guerrero-Kommritz J, Błażewicz-Paszkowycz M (2004). New species of *Tanaella* (Norman & Stebbing, 1886) (Crustacea: Tanaidacea: Tanaellidae) from the deep-sea off the Antarctic and Angola Basin, with a key to the genus. Zootaxa.

[ref-6] Hansen HJ (1913). Crustacea, Malacostraca. II. IV. The order Tanaidacea. The Danish Ingolf Expedition.

[ref-7] Kudinova-Pasternak RK (1989). Tanaidaceés abyssales (Crustacea, Tanaidacea) des parties nord-est et centrale de l ’Ocean Indien (d’ après des matériaux de l’ expedition Française “Safari-II”) 2 Sous-ordre Tanaidomorpha. Zoologicheskii Zhurnal.

[ref-8] Lang K (1968). Deep-sea Tanaidacea. Galathea Report.

[ref-9] Larsen K (2003). Proposed new standardized anatomical terminology for the Tanaidacea (Peracarida). Journal of Crustacean Biology.

[ref-10] Larsen K (2005). Deep-sea Tanaidacea (Peracarida) from the Gulf of Mexico.

[ref-11] Larsen K, Araújo-Silva CL, Coelho PA (2009). Tanaidacea from Brazil. I. The family Tanaellidae (Larsen & Wilson, 2002). Zootaxa.

[ref-12] Larsen K, Heard RW (2004). Revision of the genus *Tanaella* (Crustacea: Tanaidacea). Journal of Natural History.

[ref-13] Larsen K, Shimomura M (2007). Tanaidacea (Crustacea: Peracarida) from Japan. II. Tanaidomorpha from the East China Sea, the West Pacific Ocean, and the Nansei Islands. Zootaxa.

[ref-14] Larsen K, Wilson GDF (2002). Tanaidacean phylogeny. The first step: The superfamily Paratanaidoidea. Journal of Zoological Systematics and Evolutionary Research.

[ref-15] Morales-Núñez AG, Ardila NE (2018). Primer reporte de la familia Tanaellidae Larsen & Wilson, 2002 (Crustacea: Tanaidacea: Tanaidomorpha) en aguas del mar Caribe Colombiano.

[ref-16] Morales-Núñez AG, Morales-Ruiz C, Ardila NE (2017). First record of the family Sphyrapodidae Guţu, 1980 with the description of a new species of *Sphyrapus* from the Colombian Caribbean. PeerJ.

[ref-17] Norman AM, Stebbing TRR (1886). On the Crustacea Isopoda of the ‘Lightning’, ‘Porcupine’, and ‘Valorous’ Expeditions. Transactions of the Zoological Society of London.

[ref-18] Sieg J, Korniker LS (1986). Crustacea Tanaidacea of the Antarctic and the Subantarctic. 1 On material collected at Tierra del Fuego, Isla de los Estados, and the west coast of the Antarctic Peninsula. Biology of the Antarctic seas 18.

